# Purification and characterization of a new β-lactamase OXA-205 from *Pseudomonas aeruginosa*

**DOI:** 10.1186/s12941-015-0113-1

**Published:** 2015-11-26

**Authors:** R. Krasauskas, D. Labeikytė, A. Markuckas, J. Povilonis, J. Armalytė, R. Plančiūnienė, P. Kavaliauskas, E. Sužiedėlienė

**Affiliations:** Department of Biochemistry and Molecular Biology, Faculty of Natural Sciences, Vilnius University, M. K. Čiurlionio 21/27, 03101 Vilnius, Lithuania; Thermo Fisher Scientific Baltics, V. A. Graičiūno. 8, 02241 Vilnius, Lithuania; Institute of Microbiology and Virology, Lithuanian University of Health Sciences, Tilžės 18, 47181 Kaunas, Lithuania

**Keywords:** *Pseudomonas*, Integron, β-Lactamase, OXA

## Abstract

**Background:**

We have identified a novel class 1 integron (1503 bp), named *In671* in a clinical *Pseudomonas aeruginosa* isolate. Integron sequence analysis revealed two gene cassettes, one coding for a new OXA-type β-lactamase designated as OXA-205 and the other coding for the *aadB* gene that is responsible for aminoglycoside resistance. The 266 amino acid sequence of OXA-205 revealed that this β-lactamase belongs to the Ambler class D showing highest sequence homology to the OXA-2 sub-lineage. Our objective was to purify and characterize β-lactamase OXA-205.

**Methods:**

*Escherichia coli* cells were transformed with a plasmid containing cloned *bla*_*OXA*-*205*_ gene from *P. aeruginosa*. Purification of overproduced OXA-205 consisted of a single ion-exchange chromatography step. SDS-PAGE and isoelectric focusing were performed to determine the molecular mass and pI, respectively. Size-exclusion chromatography was undertaken to determine the OXA-205 oligomerization state. Substrate hydrolysis reactions were employed to assess enzyme kinetic parameters.

**Results:**

Purification of OXA-205 yielded the enzyme with >95 % purity (as verified by SDS-PAGE). Approximate yield of the protein was estimated to be 20 mg per liter of culture. OXA-205 had a pI at 8.1, molecular mass of 26 kDa and a monomeric native structure. Kinetic analysis revealed that OXA-205 hydrolyzed narrow spectrum substrates, including ampicillin, carbenicillin, oxacillin, penicillin G, cefazolin and cefuroxime. Additionally, we observed a substrate inhibition profile towards carbenicillin and oxacillin, but not with ampicillin or penicillin G. Our results also show that OXA-205 conferred unusually high (among class D β-lactamases) resistance towards inhibition by NaCl.

**Conclusions:**

OXA-205 can be considered a narrow spectrum monomeric β-lactamase that demonstrates unusually high resistance profile towards inhibition by NaCl.

**Electronic supplementary material:**

The online version of this article (doi:10.1186/s12941-015-0113-1) contains supplementary material, which is available to authorized users.

## Background

β-Lactamases are enzymes that are capable of degrading β-lactam antibiotics via hydrolysis of the amide bond in their β-lactam ring [1]. Today, these enzymes are of great concern for being directly or indirectly responsible for most of the multi-drug resistance observed in gram-negative bacteria in both hospital and community isolates [1, 2].

OXA-type β-lactamases are distinguished by their ability to hydrolyze cloxacillin or oxacillin at a 50 % higher rate than that of penicillin G and are inhibited by NaCl [3]. According to the functional classification, OXA-type enzymes are classified in group 2d which is further subdivided into 2 subgroups: 2de (enzymes that are unable to hydrolyze carbapenems) and 2df (enzymes with carbapenem-hydrolyzing activities) [3]. Structural classification of β-lactamases according to the Ambler assigns OXA enzymes to the D class, which includes a great number of diverse enzymes [4, 5]. However, it seems that all of them have a conserved carboxylated lysine residue in the active site [6]. Due to a great number of OXA variants and in some cases weak sequence similarity among these enzymes, data concerning their biochemical characteristics are needed to fully understand them.

In this paper, we report biochemical characteristics, substrate hydrolysis and inhibition profiles of OXA-type β-lactamase, designated as OXA-205 from a *Pseudomonas aeruginosa* imipenem resistant clinical isolate from Lithuania.

## Methods

### Bacterial isolates

*Pseudomonas aeruginosa* P16 was recovered from an eye infection of a hospitalized patient in Lithuanian University of Health Sciences Kauno Klinikos Hospital. Species identification of *P. aeruginosa* P16 isolate was performed with the automated microbiology system Phoenix™ (BD) and confirmed by PCR using primers PA-SS-F (5′-GGGGGATCTTCGGACCTCA-3′) and PA-SS-R (5′-TCCTTAGAGTGCCCACCCG-3′) [7] and reference strain *P. aeruginosa* ATCC 27853. For plasmid transfer, OXA-205 cloning and expression, *E. coli* strains JM107 (used as the primary host for recombinant plasmids), DH5α and BL21 (DE3) were used.

### Antimicrobial susceptibility testing

Antibiotic susceptibility for *P. aeruginosa* P16, *E. coli* DH5α harboring an empty vector and vector expressing OXA-205 was determined in Mueller–Hinton broth (Liofilchem), using a final inoculum of 5 × 10^5^ CFU/ml. Antibiotics were obtained from Trek. Testing was performed by the broth microdilution method, as described in the Clinical and Laboratory Standards Institute guidelines [8].

### Genetic manipulations

The molecular biology tools were purchased from Thermo Fisher Scientific Baltics and used as recommended by the manufacturer. Primers were purchased from Metabion. To clone 801 bp *bla*_OXA-205_ gene, the DNA was amplified using primers 205clF6 (5′-GTTGGAATTCATTAAAGAGGAGAAATTAAGCATGGCAATCCGATTCCTCACC-3′) and 205clR3 (5′-GCCCGGATCCAAGCAGACTTGACCTGA-3′). The former contained fragment of the vector sequence upstream from the specific part to the beginning of *bla*_*OXA*-*205*_ so that the insert would be cloned in frame. The latter was specific to the 3′ conserved segment of class 1 integrons [9]. For cloning purposes, *Eco*RI and *Bam*HI restriction sites were introduced into the primers (underlined), respectively. The obtained fragment was digested with *Eco*RI and *Bam*HI and inserted between the *Eco*RI and *Bam*HI sites of pUHE 25-2(cat) plasmid [10], resulting in plasmid pUHEcat-OXA-205 which was introduced into *E. coli* DH5α. For the purification of OXA-205, the gene without signal peptide coding region was amplified with primers 205clF5 (5′-GTTGTCTAGAAATAATTTTGTTTAACTTTAAGAAGGAGATATACCATGCAAGAACACGTGGTAGTCCG-3′), containing *Xba*I restriction site (underlined) and 205clR2 (5′-GCCCGAATTCAAGCAGACTTGACCTGA-3′). The latter was identical to the primer 205clR3, except it had *Eco*RI restriction site. The amplified fragment was digested with *Xba*I and *Eco*RI and inserted between the *Xba*I and *Eco*RI sites of pET-28b, resulting in plasmid pET-OXA-205 which was introduced into *E. coli* BL21 (DE3). *P. aeruginosa* P16 genomic DNA was used as the template. pUHEcat-OXA-205 and pET-OXA-205 transformants were selected on chloramphenicol (33 μg/ml) or kanamycin (60 μg/ml) containing LB agar plates, respectively. The accuracy of the cloned DNA inserts was verified by confirmatory sequencing (Macrogen).

### Production and purification of OXA-205

*E. coli* BL21(DE3) cells containing pET-OXA-205 plasmid were grown overnight at 37 °C in LB medium containing kanamycin (60 µg/ml). Culture was diluted 1:100 with fresh LB medium, containing kanamycin (60 µg/ml), grown at 28 °C to an A_600_ = 0.6 and induced with IPTG (Thermo Fisher Scientific Baltics) (final concentration of 1 mM) for 18 h. After induction the cells were harvested by centrifugation at 6000 × g for 15 min at 4 °C, resuspended in 50 mM Tris-H_2_SO_4_ (pH 7.4) buffer and disrupted by sonication. Debris was eliminated by centrifugation at 12000 × g for 30 min at 4 °C. The extract was filtered through a 0.22 µm membrane filter and then loaded at 2 ml/min on anion-exchange column HiLoad 16/10 Q Sepharose HP (GE Healthcare), previously equilibrated with 50 mM Tris-H_2_SO_4_ (pH 7.4).The purified OXA-205 was eluted in the flow-through fraction. HiTrap Desalting column (GE Healthcare) was used for buffer exchange using a 100 mM sodium phosphate buffer (pH 7.0) as an eluent. The fractions of the purified enzyme were stored at −80 °C. All chromatography steps were performed using ÄKTA FPLC system (GE Healthcare).

### Protein analysis

The purity of each chromatography step was determined by SDS-PAGE. Protein concentration was assayed by Bradford method using Roti–Quant kit (Roth) with bovine serum albumin (BSA) as a standard. The oligomerization state of OXA-205 was determined as described previously [11], except the Superose 12 10/300 GL column, flow rate of 0.5 ml/min and 60 µg of the purified protein were used. The column was calibrated with a mixture containing BSA (67 kDa), ovalbumin (43 kDa), chymotrypsinogen A (25 kDa), and RNaseA (13.7 kDa) (GE Healthcare) using the same flow rate conditions as mentioned above. Isoelectric focusing (IEF) was performed with IPG gel strips (pH 3–10) with Multiphor II device (GE Healthcare) according to the manufacturer’s protocol. After focusing, β-lactamase bands were detected by overlaying the strips with 0.5 mM nitrocefin. The pI values were determined and compared to those from molecular IEF standards (Bio-Rad).

### Determination of kinetic parameters

All kinetic measurements were performed at room temperature in 100 mM sodium phosphate buffer (pH 7.0) supplemented with 50 mM NaHCO_3_ and 0.2 mg/ml BSA in a total volume of 500 μl, unless specified otherwise. The variations in absorbance were measured using Genesys 10S UV–vis spectrophotometer (Thermo Scientific). The wavelengths and changes in extinction coefficients used in the spectrophotometric assays were ε_486_ = 20,500 M^−1^ cm^−1^ with nitrocefin (Calbiochem). For carbapenems and other substrates, parameters were as in [12, 11, 13], respectively. The values of kinetic parameters (K_m_ and k_cat_) were determined by measuring the initial rate (*v*) of hydrolysis of various different concentrations of β-lactams and fitting the data with non-linear regression to the Michaelis–Menten equation. K_m_ values lower than 20 μM were determined as K_i_ in a competition experiments with 0.1 mM nitrocefin as the reporter substrate [14].

For substrates that at high concentrations demonstrated substrate inhibition, data was fitted using kinetic model (Eq. 1) described by LiCata and Allewell [15].

1 $$v = \frac{{V_{max} + V_{i} \left( {{{\left[ S \right]^{x} } \mathord{\left/ {\vphantom {{\left[ S \right]^{x} } {K_{i}^{x} }}} \right. \kern-0pt} {K_{i}^{x} }}} \right)}}{{1 + \left( {{{K^{{n_{H} }} } \mathord{\left/ {\vphantom {{K^{{n_{H} }} } {\left[ S \right]^{{n_{H} }} }}} \right. \kern-0pt} {\left[ S \right]^{{n_{H} }} }}} \right) + \left( {{{\left[ S \right]^{x} } \mathord{\left/ {\vphantom {{\left[ S \right]^{x} } {K_{i}^{x} }}} \right. \kern-0pt} {K_{i}^{x} }}} \right)}}$$

where V_max_ and V_i_ correspond to the catalytic constants k_cat_ and k_cat(i)_, respectively. K is the Michaelis constant, K_i_ is the inhibition constant, [S] is the substrate concentration, *v* is the initial velocity. Exponents n_H_ and x are the Hill’s coefficients allowing for cooperativity of substrate hydrolysis and inhibition modes, respectively. In the case of complete inhibition (V_i_ = 0), the Eq. 1 reduces to the Eq. 2.

2 $$v = \frac{{V_{max} }}{{1 + \left( {{{K^{{n_{H} }} } \mathord{\left/ {\vphantom {{K^{{n_{H} }} } {\left[ S \right]^{{n_{H} }} }}} \right. \kern-0pt} {\left[ S \right]^{{n_{H} }} }}} \right) + \left( {{{\left[ S \right]^{x} } \mathord{\left/ {\vphantom {{\left[ S \right]^{x} } {K_{i}^{x} }}} \right. \kern-0pt} {K_{i}^{x} }}} \right)}}$$

The inhibitory concentration of NaCl that reduced the hydrolysis rate of substrate by 50 % (IC_50_) was assayed using 200 μM of ampicillin as the reporter substrate, under conditions in which OXA-205 was preincubated with various concentrations of salt for 3 min before the addition of the substrate.

All data fitting in this work was performed using the open source QtiPlot software [16]. Specific activity of OXA-205 was defined as the amount (unit of enzyme) that hydrolyzed 1 µmol of nitrocefin/minute/milligram of protein.

### Bioinformatic analysis

NCBI BLASTP was used to determine sequence similarities. Multiple amino acid sequence alignments were generated with the Cobalt alignment tool [17]. Sequence alignments and secondary structure information using OXA-46 structure as the reference (PDB:3IF6) were rendered using ESPript online server [18]. Theoretical calculation of protein molecular mass and pI was carried out using the software available at the ExPASy proteomic server (http://www.expasy.org/). Theoretical prediction of the leader peptide size was carried out at with the SignalP 3.0 [19]. OXA-205 model based on OXA-46 structure (PDB:3IF6) was created using SWISS-MODEL online tool [20, 21].

## Results and discussion

### Characterization of integron-encoded OXA-type β-lactamase from the *P. aeruginosa* P16 isolate

During the search for class 1and 2 integrons of a total of 111 MDR *P. aeruginosa* isolates, obtained from various clinical specimens collected in six regional hospitals in Lithuania during the period of 2005–2007, a novel class 1 integron (1503 bp), named *In671* was identified in multidrug-resistant *P. aeruginosa* isolate P16, recovered from an eye infection of a patient of Lithuanian University of Health Sciences Kauno Klinikos Hospital [22]. Antimicrobial susceptibility testing showed that the isolate was resistant to almost all antibiotics that were tested (Table 1).

**Table 1:** MIC of *E. coli* DH5α (pUHEcat-OXA-205) in comparison with *E. coli* DH5α (pUHE 25-2(cat)) and *P. aeruginosa* P16

Antimicrobial	MIC (μg/ml) for		
	*P. aeruginosa* P16	DH5α (pUHE 25-2(cat))	DH5α (pUHEcat-OXA205)
Ampicillin	512	1	512
Aztreonam	4	0.06	0.06
Cefazolin	>2048	2	16
Cefepime	2	0.02	0.03
Cefotetan	>32	0.13	4
Ceftriaxone	16	0.06	0.03
Ceftazidime	2	0.06	0.06
Cefoxitin	>64	8	8
Cefuroxime	1024	2	2
Penicillin G	2048	16	1024
Imipenem	16	0.25	0.5
Meropenem	8	0.03	0.06
Piperacillin	8	0.5	32
Cefpodoxime	>32	0.5	0.5
Carbenicillin	256	4	2048
Oxacillin	2048	128	256
Cefotaxime	16	0.03	0.03

**Fig. 1:**
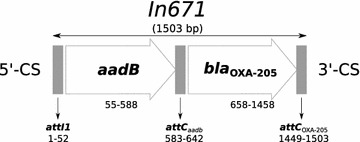
Structure of 1503 bp gene cassette array of In671 integron [GenBank:JF800667.1]. Gene cassettes are represented by *flat empty arrows*; putative elements are represented by *filled boxes*. The 5′-CS and 3′-CS show the orientation of the integron. The *thin arrows* pointing downwards show recombination sites a*ttI,* a*ttC*_*aadb*_, and a*ttC*_*OXA*-*205*_, respectively. *Numbers* mark the sequenced length of the integron and the position of each of the genes

**Fig. 2:**
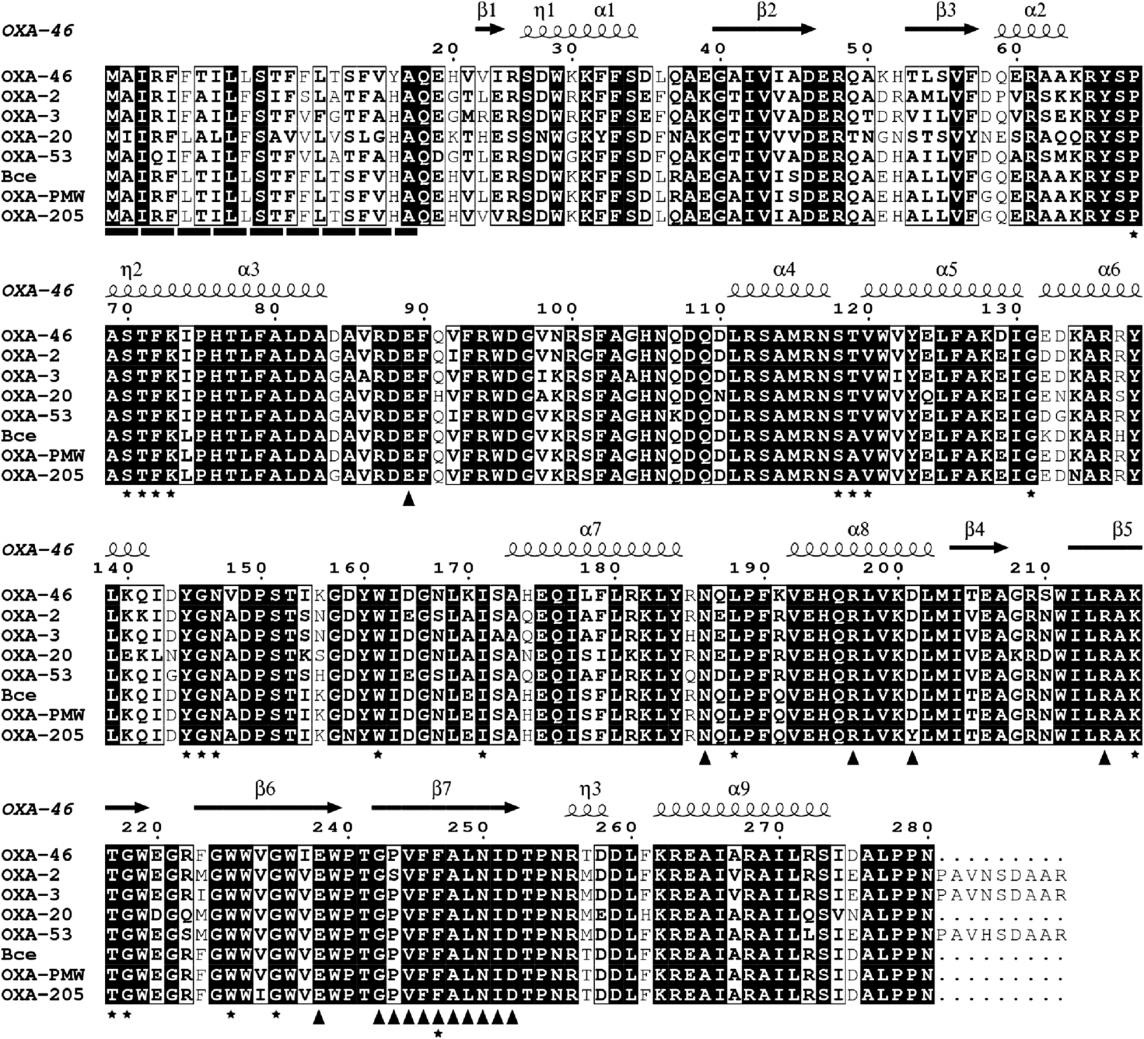
Amino acid sequence comparison of OXA-205 with the representatives of the OXA-2 lineage. Sequences of OXA-46 [GenBank:AAN63499.1] OXA-2 [GenBank:CAC82805.1]; OXA-3 [GenBank:AAC41449.1]; OXA-20 [GenBank:CAA30246.1]; OXA-53 [GenBank:AAP43641.1]; Bce (OXA-119), an OXA-type enzyme from *Burkholderia cepacia* clinical isolate from Ireland [GenBank:AAK55330.1]; OXA-PMW (OXA-118), an OXA-type enzyme encoded by a plasmid from an unidentified bacterium from a wastewater treatment plant in Germany [GenBank:AAN41427.1) are shown. The structural elements of chain C of OXA-46 enzyme (PDB:3IF6) are shown above the sequence: α, α-helices; β, β-strands; η, 3/10-helix. Predicted 21-amino acid N-terminal signal peptide is marked by *black rectangles* below the alignment. Identical residues are shaded in *black*. *Black bald characters* mark similar residues. Typical conserved motifs of class D enzymes are marked by the *asterisk* below the alignment [4]. Residues that are predicted to be involved in the dimerisation are shown by *triangles*. Residues are numbered according to class D β-lactamase (DBL) numbering [25]

Analysis of the novel 1503 bp gene cassette array of the integron revealed two gene cassettes, one coding *aadB* gene that is responsible for aminoglycoside resistance and the other coding for a new OXA-type β-lactamase, which was designated as OXA-205 (Fig. 1). Within the deduced 266 amino acid sequence all the conserved motifs typical for class D enzymes were found, namely, 70STFK73 [23], 118SXV120 [23], 144YGN146 [23], 164 W [24], 216KTG218 [23], 68P [4], 131G [4], 171I [4], 188L [4], 232 W [4], 235G [4], 247F [4] (residues are numbered according to class D β-lactamase (DBL) numbering scheme [25]) (Fig. 2). Fully matured protein (after the removal of the predicted 21 N-terminal amino acid signal peptide) is predicted to have a molecular weight (Mw) of 28,455 Da.

Comparison of amino acid sequence of OXA-205 with other class D β-lactamases revealed that it belonged to the OXA-2 sub-lineage [11] showing the highest sequence similarity to two OXA-type enzymes: OXA-118 and OXA-119, which were also found in integrons. The first one was found in uncultured bacteria from a wastewater plant and the other in a clinical *Burkholderia cepacia* isolate (97 and 96 % sequence identity, respectively) [11, 22]. OXA-205 exhibited 92 % sequence identity with the well-characterized narrow spectrum class D β-lactamase OXA-46 [11]. The remaining OXA-2 sub-linage enzymes (OXA-2, OXA-3, OXA-20, OXA-53) showed 80 % sequence similarities to OXA-205. Although *P. aeruginosa* isolate P16 was resistant to imipenem, OXA-205 sequence shared a weak similarity with carbapenem-hydrolyzing class D β-lactamases (<40 % sequence identity with OXA-23 group, OXA-48 and OXA-58 group enzymes).

### Antimicrobial susceptibility testing

*E. coli* DH5α strain was transformed with the plasmid pUHEcat-OXA-205 and compared against *E. coli* containing empty the vector pUHE 25-2(cat) in terms of susceptibility to various of β-lactams (Table 1).

The former strain demonstrated increased resistance to penicillins (ampicillin, penicillin G, piperacillin and carbenicillin), cefazolin and cefotetan. Interestingly, the OXA-205 expressing strain showed no significant increase in the resistance against oxacillin, even though this β-lactam was efficiently hydrolyzed by the purified enzyme (see below). Susceptibility of *E. coli* DH5α (pUHEcat-OXA-205) to aztreonam, cephalosporins (cefuroxime, cefoxitin, ceftazidime, ceftriaxone, cefotaxime, cefpodoxime and cefepime) and carbapenems (imipenem and meropenem) were unaffected. The obtained MIC results data allow for the conclusion that OXA-205, when expressed in *E. coli* DH5α, confers resistance against narrow spectrum β-lactams.

### Expression and biochemical properties of OXA-205

After over-expression and purification, we obtained approximately 20 mg of OXA-205 per liter of culture, with the specific activity of ~60 U/mg (measured against nitrocefin). SDS-PAGE revealed that the purified enzyme was >95 % homogeneous (Additional file 1: Figure S1). The produced β-lactamase had a pI of 8.1 and molecular weight of ~26 kDa (as judged from SDS-PAGE analysis using Fiji open source software [26]).

In order to determine the oligomerization state of OXA-205, size exclusion chromatography was undertaken. The approximate molecular mass was estimated to be 25 kDa, suggesting a monomeric native form of OXA-205. This result was quite unexpected, because dimerization had been observed among some of the D class β-lactamases, including OXA-10 [23]; OXA-13 [27]; OXA-29 [28] and, most importantly, among the three representatives of OXA-2 sub-lineage [11], namely OXA-46 [11, 29]; OXA-3 [30] and OXA-2 itself [30].

**Fig. 3:**
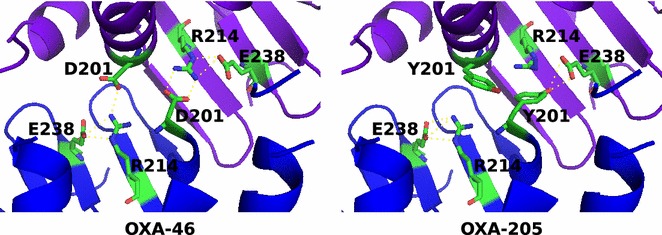
Molecular model of OXA-205 based on OXA-46 quaternary structure. D201, R210 and E238 residues forms H-bonds between different subunits in OXA-46 while D201Y substitution in OXA-205 model fails to make H-bonds with R214 residue in different subunit. Substitution also introduces clashes (data not shown). *Blue* and *purple* colors of cartoon ribbons denotes different subunits. Aforementioned residues (along with E89, N186, R197 and β7 strand strand in C-terminal end) are thought to be responsible for the formation of dimeric structure in divalent metal ions independent way [23, 29]. GMQE and QMEAN4 values of the model are 0.71 and −2.13, respectively. Model was created using SWISS-MODEL tool [20, 21] (OXA-46 assembly No. 2 from Protein data bank in Europe (PDB:3IF6) was used as a template) and visualized with the open source software PyMol (v.1.7.1.2)

It is known that at least two enzymes, OXA-29 and OXA-46, form dimers independently of divalent cations [11, 28, 29]. The solved quaternary structure of the OXA-46 revealed several amino acid residues important for the cation-independent inter-subunit interactions. These include (according to DBL numbering): β7 strand; Glu89, Asn186, Arg197, Asp201, Arg214 [29], and Glu238 [23]. Interestingly, after a close inspection of OXA-205 sequence we noticed Asp201 substitution by Tyr201. It has been proposed that Asp201 and Arg214 might form a salt bridge which could replace the cation site leading to a dimeric cation-independent structure [23, 31]. We speculate that this could be one of the main reasons why OXA-205 did not dimerize. Indeed, the model of OXA-205 based on the OXA-46 protein quaternary structure showed lack of interactions between the Tyr201and the Arg214 of OXA-205 (Fig. 3). However, site-directed mutagenesis is needed to fully support this hypothesis.

### Kinetic parameters of OXA-205

As expected, OXA-205 efficiently hydrolyzed penicillins and 1^st^ generation of cephalosporins, including ampicillin, carbenicillin, oxacillin, penicillin G and cefazolin (Table 2). Penicillin G was the best substrate that showed the highest catalytic efficiency (*k*_*cat*_*/K*_*m*_) with the value of 1.68 × 10^7^ M^−1^ s^−1^. Efficiency values for other substrates were similar to those reported for OXA-46 [11]. Interestingly, at high concentrations of carbenicillin and oxacillin (starting at approximately 2 mM and 1.6 mM for carbenicillin and oxacillin, respectively) we observed substrate inhibition kinetics. In order to measure values of constants accurately, we fitted data to the Eq. 2 because the inhibition profile demonstrated complete inhibition (Additional file 2: Figure S2). As already mentioned, the calculated efficiency values were similar to the already reported ones for OXA-46, however OXA-205 efficiency towards oxacillin, compared to OXA-2, was more than one order of magnitude lower [12]. Also, we obtained Hills coefficients (n = 2.1 ± 0.2 and x = 4.9 ± 0.7 for carbenicillin and n = 1.1 ± 0.2 and x = 3.6 ± 0.7 for oxacillin) which indicated cooperative effects in both the hydrolysis and inhibition by carbenicillin, while only inhibition by oxacillin demonstrated cooperative manner. To the best of our knowledge, substrate inhibition is not unique among β-lactamases, however it seems that it is the first example among non-metallo-β-lactamases [32, 33]. Currently, the exact mechanism of substrate inhibition for β-lactamases is unknown but it was suggested that it might be linked to the bulky aromatic ring substitutions in some of the antibiotics and the peculiarities of the active sites of β-lactamases [33].

**Table 2:** Kinetic parameters determined with the purified OXA-205 β-lactamase

Substrate	*K*_*m*_ (μM)	*k*_*cat*_ (s^−1^)	*k*_*cat*_ */K*_*m*_ (M^−1^ s^−1^)	*K*_*i*_ (μM)	*n*	*x*
Nitrocefin	6.9 ± 0.4	62.4 ± 3.3	9.09 × 10^6^			
Ampicillin	18.1 ± 0.6	36.4 ± 0.1	2.01 × 10^6^			
Carbenicillin^b^	360 ± 21	45.2 ± 1.9	1.26 × 10^5^	2887 ± 56	2.1 ± 0.2	4.9 ± 0.7
Cefazolin	4.7 ± 0.3^a^	3.9 ± 0.1	8.25 × 10^5^			
Ceftazidime	NH	NH	–			
Cefuroxime	30.4 ± 2.5	0.02 ± 0.001	6.69 × 10^2^			
Imipenem	0.02 ± 0.002^a^	0.03 ± 0.001	1.39 × 10^6^			
Meropenem	0.02 ± 0.002^a^	0.008 ± 0.0005	3.08 × 10^5^			
Oxacillin^b^	1369 ± 87	376 ± 25	2.75 × 10^5^	1869 ± 75	1.1 ± 0.1	3.6 ± 0.2
Penicillin G	2.3 ± 0.06^a^	39.1 ± 2	1.68 × 10^7^			

Reactions were carried out using the concentration of OXA-205 ranging from 2.5 to 814 nM. All values are the means of at least three different measurements

*NH* no hydrolysis observed at a substrate concentration up to 1 mM

– Not calculated

^a^Measured as an inhibition constant (K_i_) in competition experiment against nitrocefin [13]

^b^Obtained data was fitted to the Eq. 2 because substrate inhibition kinetics were observed

Also, we observed that OXA-205 influenced hydrolysis of the 2nd generation cefalosporin cefuroxime. However, a very low turnover number (*k*_*cat*_) and rather high affinity (*K*_*m*_) resulted in poor hydrolysis efficiency (6.69 × 10^2^ M^−1^ s^−1^). Lastly, we did not observe any of OXA-205 influenced hydrolysis of ceftazidime (the 3rd generation cefalosporin), suggesting that OXA-205 could be considered a narrow spectrum β-lactamase towards cefalosporins.

It has been shown that OXA-2 displays similar catalytic efficiencies towards carbapenems as some of the recognized class D carbapenemases [13]. Strikingly, our data supported this observation—OXA-205 hydrolyzed imipenem and meropenem as efficient as ampicillin and oxacillin, respectively. However, OXA-205 displayed very low turnover numbers, which were approximately one order of magnitude lower when compared to OXA-2. Nevertheless, the enzyme demonstrated a high affinity towards carbapenems. This data support the suggestion that other class D enzymes which currently are regarded as non-carbapenemases may, in fact be carbapenem-hydrolysing class D β-lactamases [12].

Inhibition by sodium chloride (NaCl) is considered one of the useful characteristics for in vitro identification of class D β-lactamases: most enzymes are fully inactivated with ≥100 mM NaCl [34]. In order to determine the pattern of susceptibility to NaCl, we performed inhibition experiments which concluded that OXA-205 was inhibited by salt at extremely high concentration—IC_50_ = 1361 ± 118 mM. Interestingly, OXA-46 also demonstrated reduced susceptibility to NaCl (IC_50_ at ~230 mM) [11]. These results suggest that low resistance to NaCl may be widespread among the enzymes belonging to the OXA-2 sub-lineage.

## Conclusion

Our study characterized a new OXA-type β-lactamase OXA-205 which displayed a monomeric oligomerization state which we speculated could at least partially was influenced by a single amino acid substitution. Our results also showed that even though OXA-205 demonstrated narrow spectrum hydrolysis profile towards penicillins and cephalosporins it also readily hydrolyzed carbapenems and conferred unusually high resistance towards inhibition by NaCl. Interestingly, carbenicillin and oxacillin, but not other penicillins demonstrated a substrate inhibition profile which could be considered as a novel feature among class D β-lactamases. All in all, our work further expands knowledge regarding molecular and biochemical heterogeneity of β-lactamases.

## Additional files

10.1186/s12941-015-0113-1 SDS-polyacrylamide gel of OXA-205 purification. Lanes: 1 – cell fraction of *E. coli* BL21 (DE3) (pET-OXA-205) before induction with 1 mM IPTG; 2–Thermo Scientific PageRuler Prestained Protein ladder (SM0671); 3 – cell fraction of *E. coli* BL21 (DE3) (pET-OXA-205) after induction with 1 mM IPTG; 4 – purified cell fraction after anion-exchange, using 10 μg of total protein; 5 – purified cell fraction after anion-exchange, using 20 μg of total protein; 6 – purified cell fraction after anion-exchange, using 30 μg of total protein; 7–purified cell fraction after anion-exchange, using 40 μg of total protein. Relative migrations of Mw marker proteins are indicated on the left.
10.1186/s12941-015-0113-1 Inhibition of OXA-205 by carbenicillin and oxacillin. The data (filled square) is plotted as initial velocity (v) versus substrate concentration for oxacillin (upper plot) and carbenicillin (lower plot). Error bars represent standard deviation from three different measures. The solid line in each plot is a fit to the data using Eq. 2 using kinetic parameters form Table 2.

## Abbreviations

Mw: molecular weight
MDR: multi-drug resistant
bp: base pairs
LB: Luria-Bertani medium
DBL: Class D β-lactamase
IC_50_: the inhibitor concentration that reduced the hydrolysis rate of substrate by 50 %
MIC: minimal inhibitory concentration
