# Supplementary material for: Purification and characterization of a new β-lactamase OXA-205 from Pseudomonas aeruginosa
Source: Ann Clin Microbiol Antimicrob. 2015 Nov 26;14:52. doi: 10.1186/s12941-015-0113-1 (PMC4661998; doi:10.1186/s12941-015-0113-1)
Supplement: Supplementary file 1 — 10.1186/s12941-015-0113-1 SDS-polyacrylamide gel of OXA-205 purification. Lanes: 1 – cell fraction of E. coli BL21 (DE3) (pET-OXA-205) before induction with 1 mM IPTG; 2–Thermo Scientific PageRuler Prestained Protein ladder (SM0671); 3 – cell fraction of E. coli BL21 (DE3) (pET-OXA-205) after induction with 1 mM IPTG; 4 – purified cell fraction after anion-exchange, using 10 μg of total protein; 5 – purified cell fraction after anion-exchange, using 20 μg of total protein; 6 – purified cell fraction after anion-exchange, using 30 μg of total protein; 7–purified cell fraction after anion-exchange, using 40 μg of total protein. Relative migrations of Mw marker proteins are indicated on the left. [file 12941_2015_113_MOESM1_ESM.pdf]

Additional files

Additional file 1 – SDS-polyacrylamide gel of OXA-205 purification

Lanes: 1 – cell fraction of *E. coli* BL21 (DE3) (pET-OXA-205) before induction with 1 mM IPTG; 2 – Thermo Scientific PageRuler Prestained Protein ladder (SM0671); 3 – cell fraction of *E. coli* BL21 (DE3) (pET-OXA-205) after induction with 1 mM IPTG; 4 – purified cell fraction after anion-exchange, using 10 μg of total protein; 5 – purified cell fraction after anion-exchange, using 20 μg of total protein; 6 – purified cell fraction after anion-exchange, using 30 μg of total protein; 7 – purified cell fraction after anion-exchange, using 40 μg of total protein. Relative migrations of Mw marker proteins are indicated on the left.

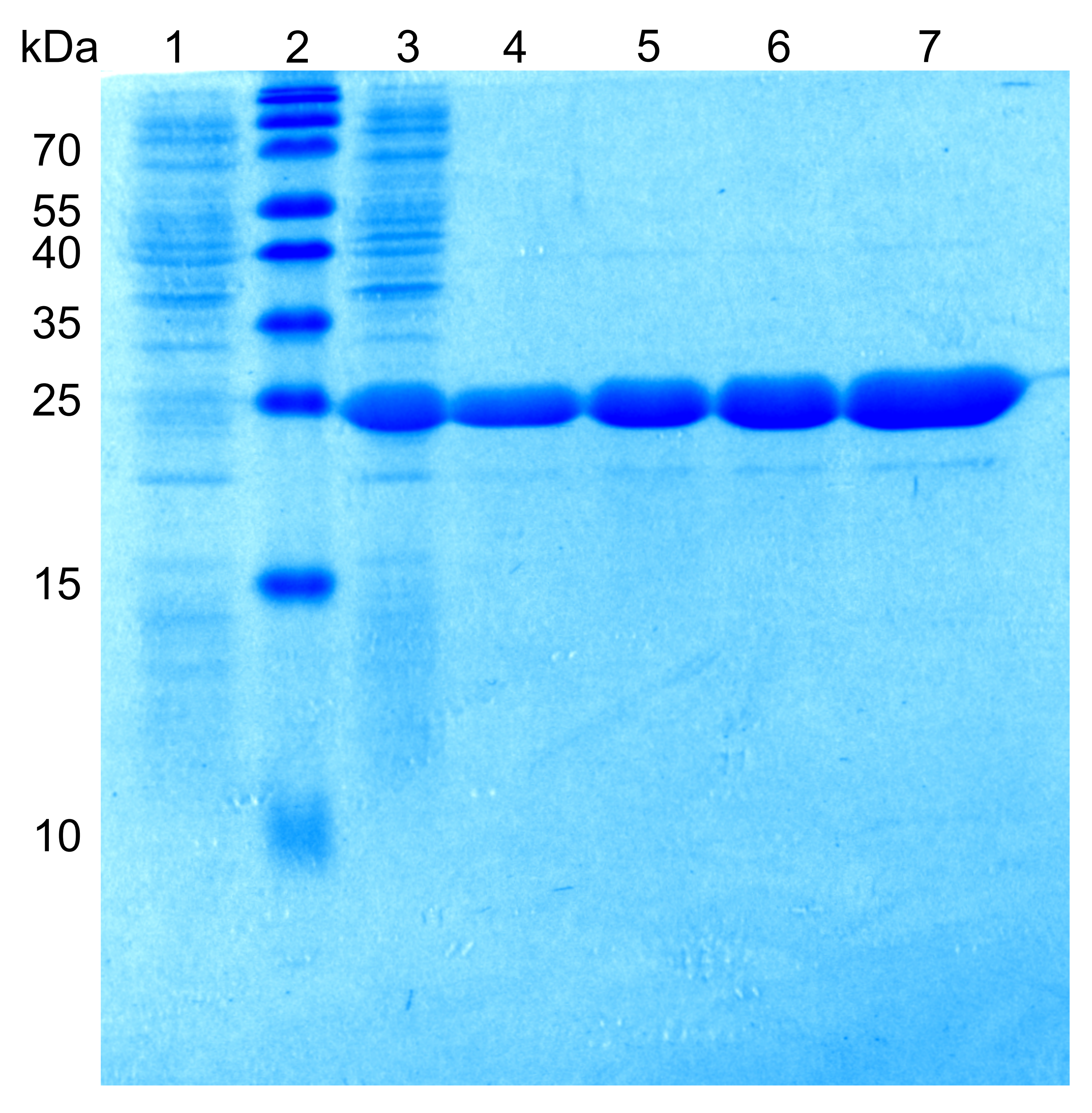
kDa
1
2
3
4
5
6
7
70
55
40
35
25
15
10
