# Supplementary material for: Purification and characterization of a new β-lactamase OXA-205 from Pseudomonas aeruginosa
Source: Ann Clin Microbiol Antimicrob. 2015 Nov 26;14:52. doi: 10.1186/s12941-015-0113-1 (PMC4661998; doi:10.1186/s12941-015-0113-1)
Supplement: Supplementary file 2 — 10.1186/s12941-015-0113-1 Inhibition of OXA-205 by carbenicillin and oxacillin. The data (filled square) is plotted as initial velocity (v) versus substrate concentration for oxacillin (upper plot) and carbenicillin (lower plot). Error bars represent standard deviation from three different measures. The solid line in each plot is a fit to the data using Eq. 2 using kinetic parameters form Table 2. [file 12941_2015_113_MOESM2_ESM.pdf]

Additional files

Additional file 2. Inhibition of OXA-205 by carbenicillin and oxacillin

The data (■) is plotted as initial velocity (*v*) versus substrate concentration for oxacillin (upper plot) and carbenicillin (lower plot). Error bars represent standard deviation from three different measures. The solid line in each plot is a fit to the data using Equation 2 using kinetic parameters form Table 2

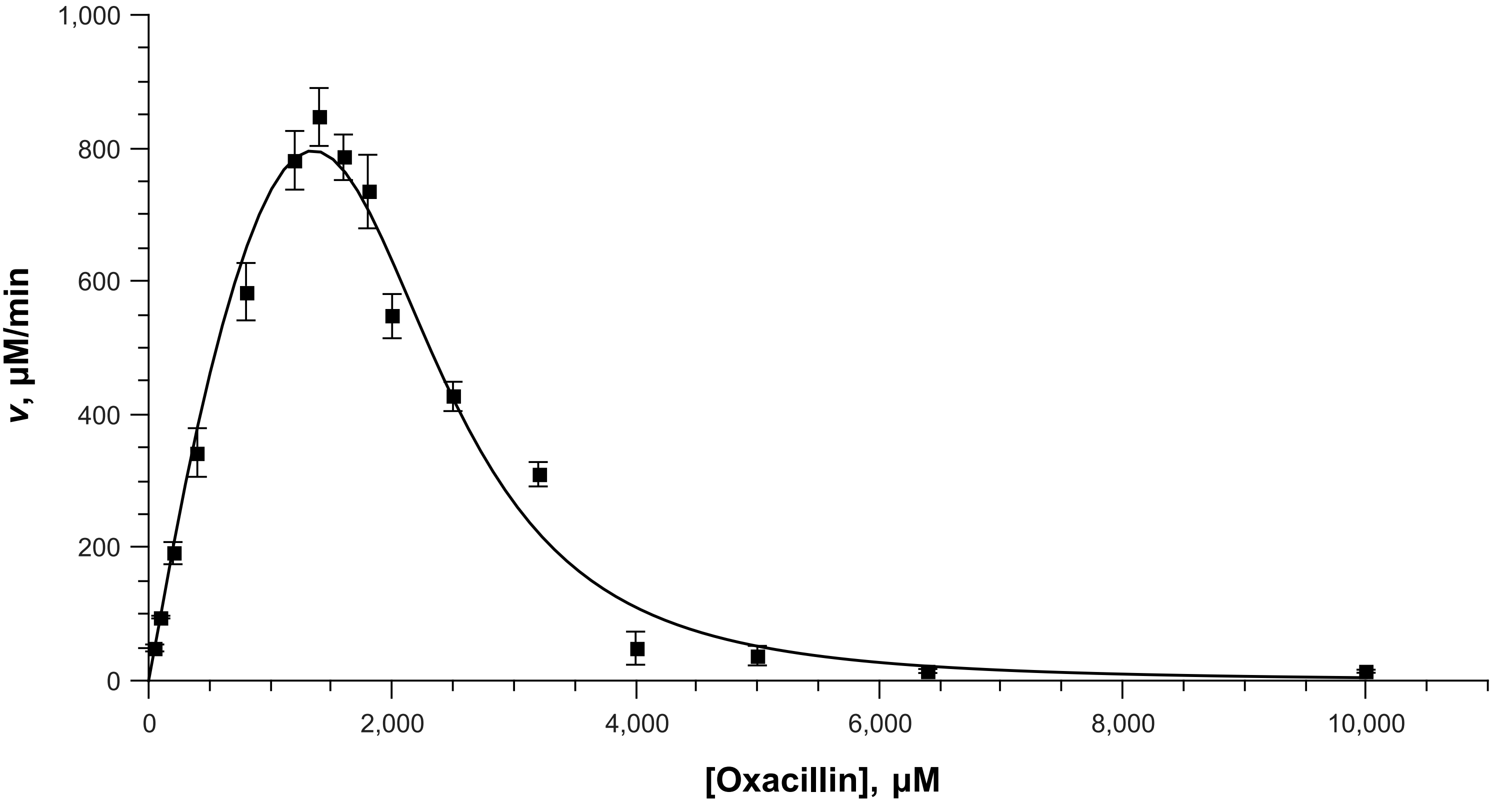
1,000
800
600
400
200
0
v, μM/min
0
2,000
4,000
6,000
8,000
10,000
[Oxacillin], μM

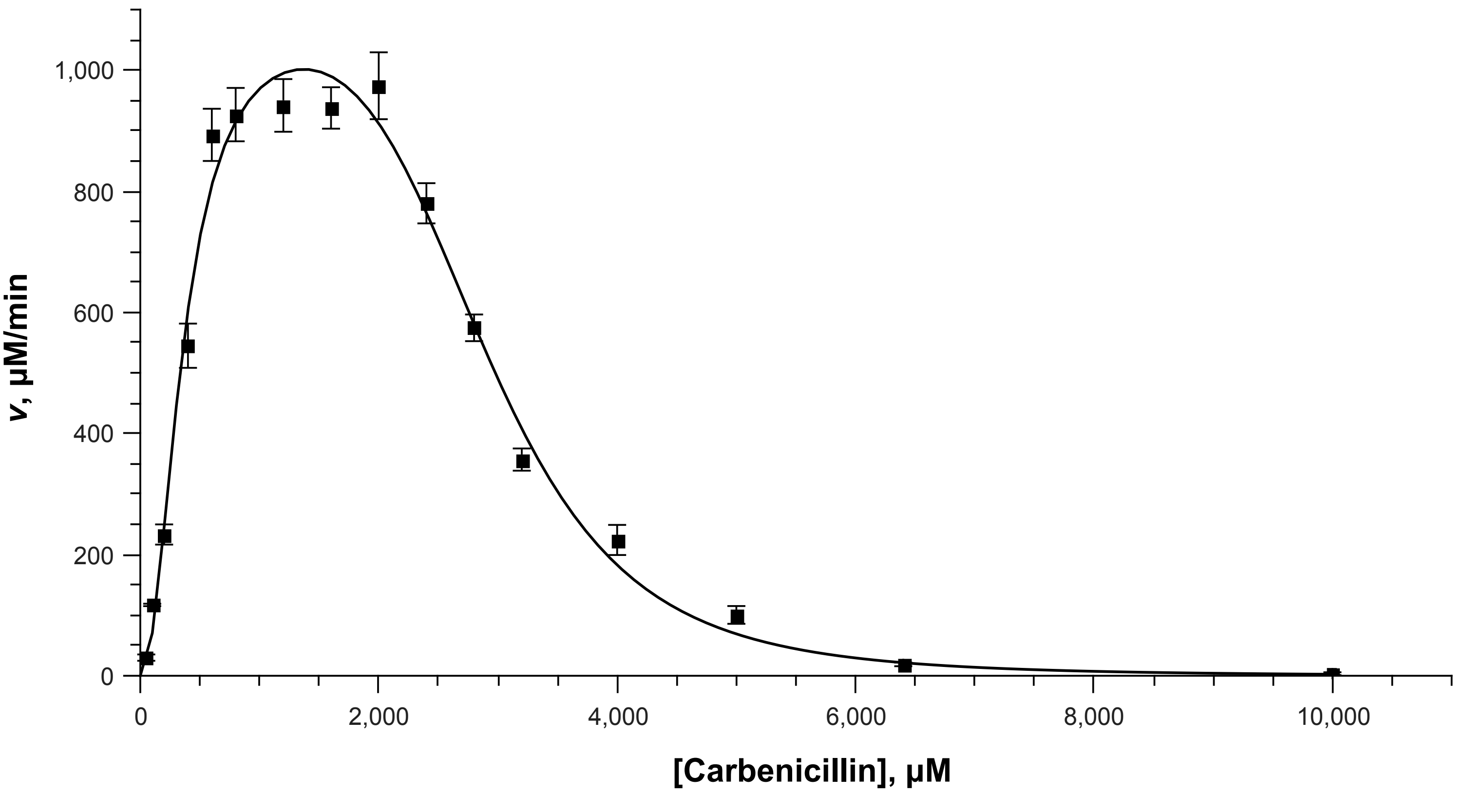
1,000
800
600
400
200
0
v, μM/min
0
2,000
4,000
6,000
8,000
10,000
[Carbenicillin], μM
